# Navigating the CT wave: multicentric chest imaging trends before, during, and after COVID-19 Pandemic

**DOI:** 10.1007/s00330-025-12030-0

**Published:** 2025-10-08

**Authors:** Thiago Lima, Natalia Saltybaeva, Tobias Gassenmaier, Lukas Ebner, Justus E. Roos

**Affiliations:** 1 Department of Radiology and Nuclear Medicine, Luzerner Kantonsspital, University Teaching and Research Hospital, University of Lucerne, Lucerne, Switzerland; 2 Faculty of Health Sciences and Medicine, University of Lucerne, Lucerne, Switzerland; 3 Department of Nuclear Medicine, Swiss Medical Network, Genolier, Switzerland; 4 Department of Diagnostic, Interventional and Pediatric Radiology, Inselspital, Bern University Hospital, University of Bern, Bern, Switzerland; 5 Beau-Site Klinik Hirslanden, Bern, Switzerland

**Keywords:** Chest examinations, CT, Trends, COVID-19

## Abstract

**Objective:**

The COVID-19 pandemic has significantly impacted healthcare practices worldwide, including the use of computed tomography (CT) in chest examinations. This study aims to analyse the trends in CT chest examinations before, during, and after the pandemic.

**Material and methods:**

Data were retrospectively collected from 10 public hospitals over a period spanning from pre-pandemic years (2019 until February 2020) through the pandemic (March 2020–April 2023) (as defined by the World Health Organisation) and into the post-pandemic phase (May 2023–December 2024). Patient exposure information from more than 240,000 CT examinations was collected and analysed using a commercial dose management system. Statistical analysis was performed with descriptive and inferential statistics employed to compare the number of CT chest examinations and patient radiation exposure across different periods.

**Results:**

The results indicate a marked increase in CT chest examinations during the pandemic, which reached a plateau and remained stable post-pandemic (*p*-value < 0.001). Importantly, the average radiation exposure per patient has decreased with the evolution of technology, indicating improved dose management practices. The results also showed a shift between protocols used for CT chest examinations during the pandemic, with the move towards more dedicated procedures.

**Conclusion:**

These findings highlight the sustained demand for CT chest examinations and the effective management of patient radiation exposure during and after the pandemic. The study underscores that those possible practices obtained during the pandemic became the norm after the end of the pandemic.

**Key Points:**

***Question*** *This study analyses trends in CT chest examinations and patient radiation exposure before, during, and after the COVID-19 pandemic*.

***Findings*** *CT chest examinations rose sharply during the pandemic, then stabilised, while average patient radiation exposure remained consistent throughout*.

***Clinical relevance*** *The findings demonstrate ongoing demand for CT chest scans and radiation management, emphasizing the need for sustainable imaging practices in future healthcare planning.*

**Graphical Abstract:**

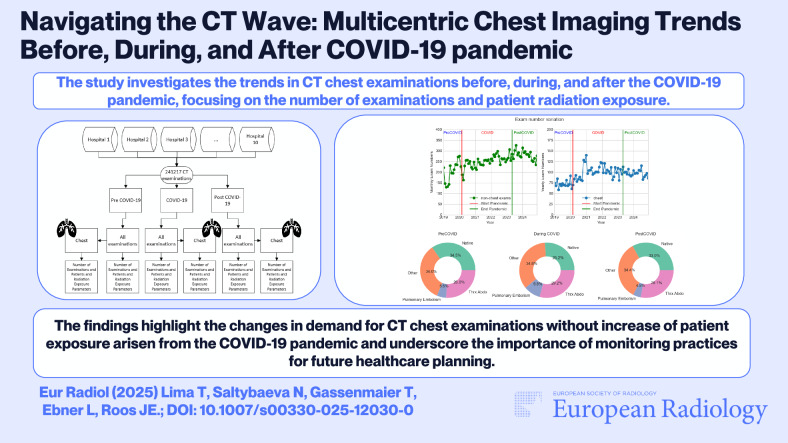

## Introduction

The COVID-19 pandemic has led to unprecedented changes in healthcare delivery, particularly in diagnostic imaging. Computed tomography (CT) has been a critical tool in the diagnosis and choice of the treatment strategy of COVID-19-related chest conditions. CT imaging provides detailed cross-sectional images of the chest, allowing for the identification of characteristic features of COVID-19 pneumonia, such as ground-glass opacities and consolidation [1–6].

The role of CT in diagnosing COVID-19 has been well-documented. Early studies indicated that CT imaging could detect chest abnormalities even in asymptomatic patients, making it a valuable tool for early diagnosis and management [7, 8]. Additionally, CT imaging has been used to monitor disease progression and assess treatment efficacy [9]. However, the surge in CT imaging during the pandemic has also led to increased concerns about radiation exposure, particularly in vulnerable populations such as the elderly and those with pre-existing health conditions [10–12].

Previous studies have shown significant growth in the use of CT imaging during the early stages of the pandemic [13–19]. However, there is limited data on the long-term trends in CT chest examinations and patient radiation exposure beyond the initial pandemic period. This study aimed to fill this gap by analyzing the trends in CT chest examinations before, during, and after the COVID-19 pandemic, with a focus on the number of examinations and patient radiation exposure. Understanding these trends is crucial for developing sustainable diagnostic imaging practices and ensuring patient safety in the long term.

## Materials and methods

Patient exposure information was collected and analyzed using a commercial dose management system (Teamplay, Siemens Healthineers) and its business intelligence platform, Insights. Data was gathered from 16 CT devices (over the period of the study, some devices were changed but for this analysis, each individual device was counted separately) across 10 hospitals over a period spanning from pre-pandemic (January 2019–February 2020) through the pandemic (March 2020–May 2023) and into the post-pandemic phase (June 2023–December 2024). The number of CT examinations, together with radiation exposure metrics such as CTDIvol and DLP, was collected. The dose metrics averaged over the patient population within each considered period, and their SD were calculated. The results were compared between the chest examinations and the total.

Descriptive statistics were used to summarize the data, and inferential statistics were employed to compare the number of CT chest examinations and patient radiation exposure across different time periods. The chi-square test was used to assess the significance of differences in the number of examinations, while the independent *t*-test was used to compare average patient radiation exposure. A *p*-value of < 0.05 was considered statistically significant. Statistical analysis was performed using Scipy (version 1.14.1) statistical package from Python.

The North and Central Switzerland Ethics Commission waived institutional review board approval for this project (Req-2025-00111), and all patient data was anonymised.

## Results

**Fig. 1:**
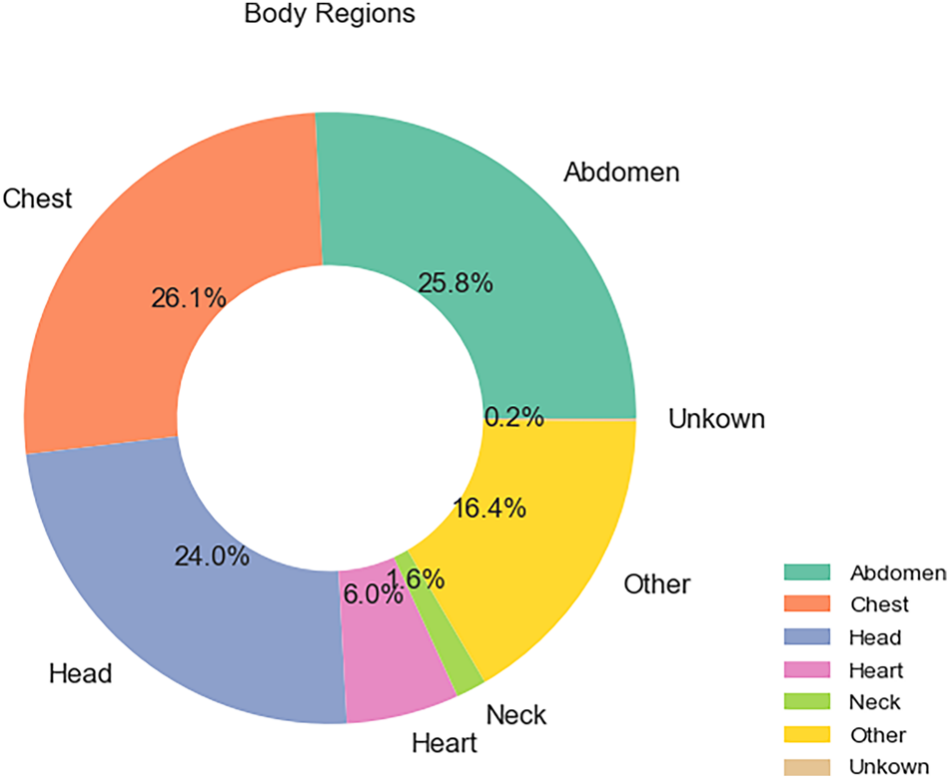
Describe the body region examinations from the 241,217 CT examinations collected in this study

A total of 241,217 CT examinations were conducted across ten hospitals during the study period. Of these, 62,981 were chest CT examinations, representing 26.1% of the total CT examinations. Figure 1 below shows the distribution of the total CT examinations per different body regions.

Table 1 describes the examination numbers for the three periods of interest: before, during and after the COVID-19 pandemic. The numbers (median, variance, standard deviation, minimum and maximum) are shown per month and aggregated (total, weight and age) for the period.

**Table 1:** All numbers are per month and split between all CT examinations and chest CT examinations. The weight and age are split by the different periods and described as mean (minimum, maximum) for all examinations

	Before pandemic		During pandemic		Post pandemic	
	All	Chest	All	Chest	All	Chest
Median	2137.5	508.5	3312.0	897.0	4362.0	1080.0
Variance	237,724.6	8717.3	196,946.7	23,351.8	103,584.0	14,032.7
STDev	487.6	93.4	443.8	152.8	321.8	118.5
min	1342	353	2216	633	3618	842
max	2939	729	4130	1222	4910	1265
Total per period	29131	6999	129590	35530	82496	20,452
Weight (kg)	75.3 (3.1, 213.4)		75.9 (2.8, 215.0)		76.1 (3.2, 227.0)	
Age (years)	61.5 (0.0, 90.0)		61.8 (0.0, 90.0)		62.1 (0.0, 91.1)	

**Fig. 2:**
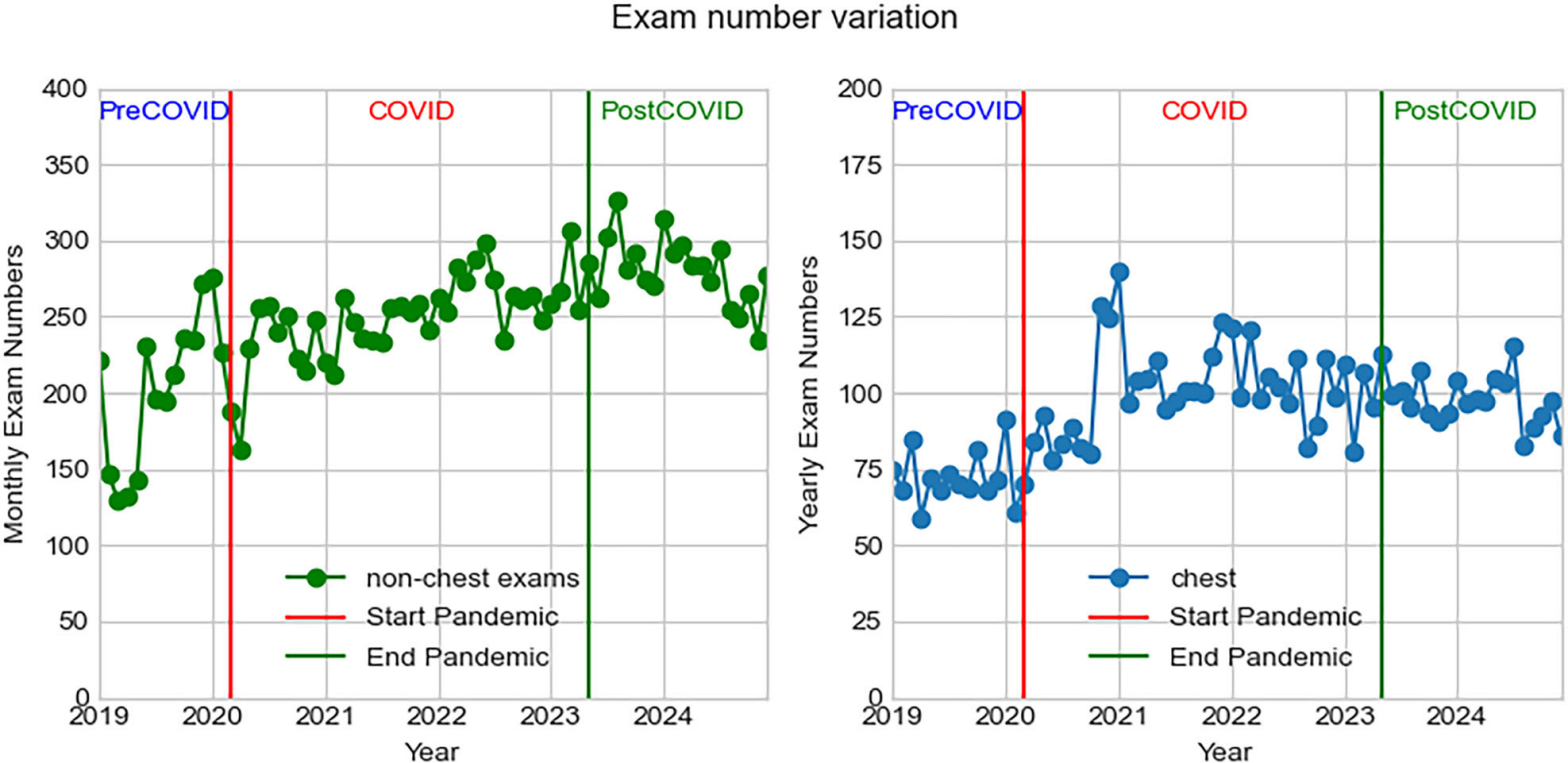
Describe the trend of CT examinations from the 241217 CT examinations collected in this study for both all regions (left figure) and only chest examinations (right)

Figure 2 shows both the monthly and yearly variation of exam numbers during the whole evaluated period.

The results showed a steady increase in all types of CT examinations from 2019 to 2024, as shown in Tables 2 and 3. Comparing the average examination numbers per year, normalized by device number, between 2024 and 2019, this represented a 58.8% increase in examinations. The amount of chest examinations also increased from an average of 636 to 1058.4 CTs between these two years, and their proportion among all CT examinations grew from 24.3% to 25.4% (*p*-value 0.33). Between 2019 and 2020, there was an increase of 50% considering the average of chest examinations per year normalized for the number of devices; this increase was statistically significant with a *p*-value < 0.001. For the subsequent period post-pandemic, in 2023, there was a slight reduction (*p*-value 0.14) in examination numbers, with an average of 935.9 chest examinations and 3868.3 for all body regions. This represents 24.2% of the examinations that have been performed in these hospitals.

**Table 2:** Exam numbers and radiation dose parameters per year for the chest examinations

Year	CTDIvol (mGy)	DLP (mGy.cm)	Scan length (cm)	Examination numbers	Number of devices	Exams per device	Proportion of total exams
2019	9.69	351.0	36.2	5724	9	636.0	24.3%
2020	9.25	352.5	38.1	8893	9	988.1	26.6%
2021	8.37	362.9	43.4	11,724	11	1062.8	29.1%
2022	8.03	353.7	44.1	11,618	11	1056.2	26.9%
2023	7.50	328.6	43.8	11,231	12	935.9	24.2%
2024	7.44	323.8	43.5	13,759	13	1058.4	25.4%

**Table 3:** Exam numbers and radiation dose parameters per year for all CT examinations

Year	CTDIvol (mGy)	DLP (mGy.cm)	Scan length (cm)	Examination numbers	Number of devices	Exams per device
2019	16.08	441.0	27.4	23,600	9	2622.2
2020	16.86	434.5	25.8	33,490	9	3721.1
2021	16.12	441.1	27.4	40,223	11	3656.6
2022	16.14	444.8	27.6	43,263	11	3933.0
2023	16.48	461.3	28.0	46,419	12	3868.3
2024	15.97	448.8	28.1	54,118	13	4162.9

The analysis revealed a significant increase in the number of CT chest examinations during the COVID-19 pandemic. This increase reached a plateau and remained stable in the post-pandemic period. The overall examination number for all CT examinations is still on the rise.

**Fig. 3:**
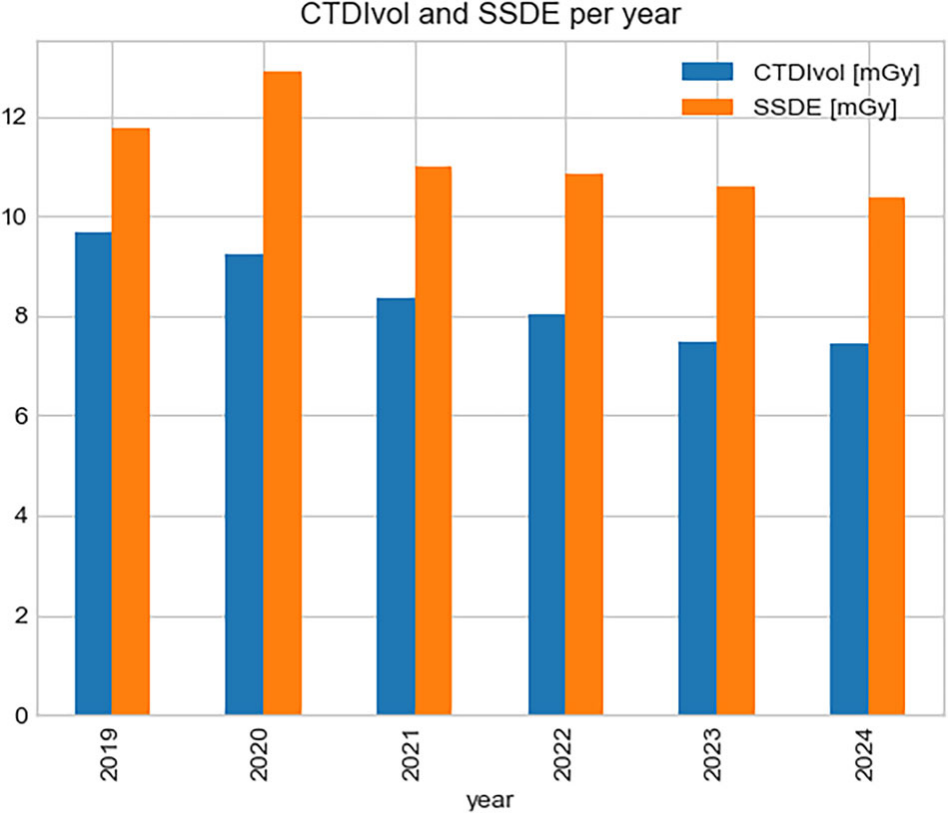
Evolution of CTDIvol and size-specific dose estimate (SSDE) for chest examinations in the evaluated period. Here is the mean CTDIvol per device per year

Notably, the average patient exposure to radiation was level throughout the evaluated period, indicating effective dose management practices. Specifically for the chest examinations, this exposure decreased with the introduction of more modern technologies and continuing optimization of protocols as shown in Fig. 3.

**Fig. 4:**
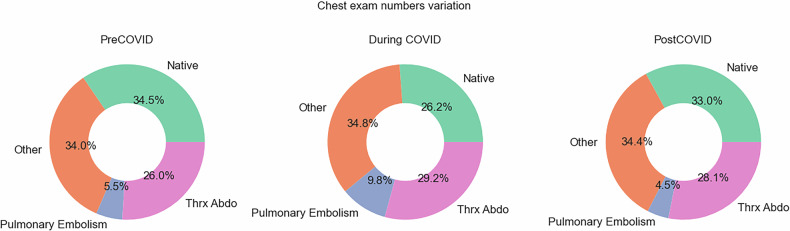
Evolution of CT chest protocols in the evaluated period

Figure 4 shows the protocol usage varied significantly before, during, and after the pandemic, reflecting different clinical needs. Before the pandemic, CT thorax–abdomen represented 26% of chest examinations, pulmonary embolism accounted for 5%, and thorax native accounted for 35%. During the COVID-19 pandemic, these representations shifted to 29% for thorax–abdomen, 10% for pulmonary embolism, and 26% for thorax native. In the post-pandemic period, thorax native increased to 33%, thorax–abdomen protocol accounted for 28%, but pulmonary embolism examinations fell to 4%. This can also be observed by the changes in scan length reported in Tables 2 and 3, while the scan length remained stable for all examinations. For chest examination, there was an increase and then reached a plateau. The average scan length for chest CT native examination, pulmonary embolism and thorax-abdomen were 36.3, 33.7 and 48.7 cm, respectively.

## Discussion

The findings of this study demonstrate the sustained demand for CT chest examinations due to the COVID-19 pandemic and the subsequent stabilization of this demand post-pandemic. These results are in line with previous studies that have highlighted the critical role of CT imaging in the diagnosis and management of COVID-19 [1–6].

## Increase in CT examinations during the pandemic

The data shown in Fig. 2 indicates a substantial increase in the number of CT examinations during the COVID-19 pandemic. This surge is particularly notable for chest CT examinations, which saw a significant rise in both absolute numbers and as a proportion of total CT examinations. This trend aligns with global observations where the demand for chest imaging increased due to the need for rapid and accurate diagnosis of COVID-19-related pulmonary complications [13–19].

Kempter et al [13] aimed to examine the impact of the COVID-19 pandemic on CT examination volumes in the emergency department, anticipating that changes encountered during the COVID-19 pandemic would subsequently influence examination volumes in the post-pandemic period. The study found that dual-region CT scans experienced the highest relative increase in monthly volume, followed by CT polytrauma, CT chest, CT head, and CT abdomen-pelvis examinations. The usage of dual-region and chest CT examinations was higher during and after the COVID-19 pandemic, compared to the control period. The study found a significant increase in dual-region CT and chest CT scans, with an 88.4% and 70.7% increase, respectively, in the post-pandemic period compared to pre-pandemic levels, while chest radiograph volumes decreased by 36.4%.

In contrast to the findings of Kempter et al, whose publication showed a continuing decrease in the chest examination numbers post-COVID, our results show a plateau in the number of CT chest examinations post-pandemic, suggesting that healthcare providers have adapted to the increased demand for diagnostic imaging and have implemented sustainable practices. One possible difference between these results can be explained by the origin of CT numbers. In their publication, the CT numbers were linked with emergency department numbers. Our results it is based on the total numbers from the radiology departments. Post-pandemic, the number of CT examinations remained elevated compared to pre-pandemic levels, although there was a slight reduction in the number of chest CT examinations compared to the pandemic period. This plateau suggests that while the immediate surge in demand driven by COVID-19 has subsided, the overall utilization of CT imaging has not returned to pre-pandemic levels. This sustained increase could be attributed to several factors, including the continued use of CT imaging for post-COVID-19 complications and other clinical indications for CT examination of the chest region (for example, new cardiac protocols).

Bonacossa de Almeida et al [14] aimed to assess the impact of the COVID-19 pandemic on the volumes of use of diagnostic imaging examinations in the Brazilian Unified Health System (SUS). They found that there was a large reduction in the use of most types of diagnostic imaging examinations in 2020 compared to 2019, with greater decreases among outpatients than inpatients. And that CT was the only modality that increased in use in 2020, driven by a much higher use of chest CT scans. The highlighted that the changes in diagnostic imaging use correlated with the worsening of the COVID-19 pandemic in Brazil, starting around March–April 2020. These findings align with the trend observed in our study, where the use of CT for chest protocols increased during the pandemic.

## Radiation exposure due to these trends

A different study aimed to determine if chest CT was excessively used during the COVID-19 pandemic, leading to increased radiation exposure, especially among young and middle-aged people [18]. They warned that chest CT was excessively used during the COVID-19 pandemic, leading to a 573% increase in radiation exposure for infectious disease indications compared to the previous year. Although this increase can be explained by the total number of examinations presented, these results can also be misleading because during the same period, there was an increase of over 500% in patient numbers, so the radiation exposure increase per examination is not on that order of magnitude. Our results in Fig. 3 showed that although the volume of CT examinations increased, the average radiation exposure was reduced. The dose reductions observed in this study can be explained by the evolution of technology (new hardware and software), when older devices were replaced by newer ones, and by continuing protocol optimization, or a mixture of both factors. An interesting finding presented by Coskun et al [18] was the differences in radiation exposure across different age groups due to the increased use of chest CT scans during the pandemic and the impact on repeated examinations.

## Evolution of chest protocols

During the pandemic, chest CT scan length increased and remained higher post-pandemic, reflecting broader indications and wider fields of view—a trend not seen with other CTs. These changes in scan length closely follow protocol shifts; for instance, the average scan length is 48 cm for Thorax–Abdomen protocols, while native and pulmonary embolism protocols average 36 cm and 33 cm, respectively.

Moreover, the findings of this study have important implications for healthcare providers and policymakers. The sustained demand for CT chest examinations highlights the need for continued investment in diagnostic imaging infrastructure and training. Additionally, the consistent patient radiation exposure observed in this study underscores the importance of implementing and maintaining effective dose management practices and the evolution of the technology. These findings provide valuable insights for healthcare providers and policymakers in planning and optimizing diagnostic imaging services in the context of ongoing and future public health challenges.

Future research should focus on further refining dose management practices and optimizing imaging protocols and practices that offer similar diagnostic benefits with lower radiation exposure. Additionally, longitudinal studies are needed to assess the long-term health outcomes of patients who underwent frequent CT imaging during the pandemic. Such research will be crucial for developing evidence-based guidelines for the use of CT imaging in the diagnosis and management of respiratory diseases, as well as monitoring of future pandemics, as highlighted in international initiatives like ZODIAC from the International Atomic Energy Agency and PRET from the World Health Organization.

## Limitations

This study has a few limitations that should be acknowledged. Firstly, the data presented are derived directly from actual DICOM data, which, while ensuring reproducibility and objectivity, may not capture all clinical nuances, for example, a patient being imaged on the wrong protocol. Secondly, the analysis of radiation exposure was conducted primarily to demonstrate overall trends in dose levels rather than to thoroughly investigate or attribute the main factors underlyin g the observed changes. As such, the study does not offer a detailed breakdown of the technological, protocol-related or patient-specific contributors to dose evolution. Finally, there was no specific assessment of the risks associated with cumulative radiation exposure resulting from multiple CT examinations. Consequently, the findings do not provide a comprehensive evaluation of long-term patient risk, which would require dedicated follow-up and risk stratification methodologies.

## Conclusion

In conclusion, the COVID-19 pandemic has led to a significant increase in the use of CT chest examinations, which reached a plateau and remained stable post-pandemic. The consistent average patient exposure to radiation highlights the effectiveness of dose management systems in maintaining patient safety. These findings underscore the importance of sustainable diagnostic imaging practices and provide valuable insights for future healthcare planning.

## Abbreviations

COVID-19: Coronavirus disease 2019
CT: Computed tomography
